# Supplementary material for: Deep Profiling of Mouse Splenic Architecture with CODEX Multiplexed Imaging
Source: Cell. 2018 Aug 9;174(4):968–981.e15. doi: 10.1016/j.cell.2018.07.010 (PMC6086938; doi:10.1016/j.cell.2018.07.010)
Supplement: Table S4. Linear Regression Model for Marker Expression Level Based on Niche and Cell Type Shows Importance of Niche, Related to Figures 4D and 4E — The overall role of the niche in defining marker expression was evaluated by constructing a linear regression model of marker expression with cell type identity and niche as two feature variables. This Excel file shows F and P values for the contribution of niche to the model. The F value is the ratio of the mean regression sum of squares for the model including just cell type to the full model including both niche and the cell type. Its value ranges zero to an arbitrarily large number. A larger F value suggests that the niche has a larger contribution in explaining the variance observed in the expression levels of each marker. The value of Pr(> F) is the p value against the null hypothesis that including the niche in the model does not improve the fit. [file mmc4.pdf]

F and Pr(>F) values for niche contribution in fitting surface marker expression with a regression model

Marker_expression~factor(celltype)+factor(niche_ID)

	F values	Pr(>F)
**B220**	394.713564	0
**CD21/35**	344.790481	0
**ERTR7**	319.034053	0
**CD90**	296.257425	0
**CD35**	275.720623	0
**F480**	196.705914	0
**CD169**	178.874821	0
**CD106**	115.018257	0
**CD19**	103.935024	0
**IgD**	88.3376641	0
**CD44**	84.3808551	0
**Ter119**	81.3449664	0
**CD4**	74.6449407	0
**CD1632**	68.9150189	0
**CD79b**	68.061559	0
**NKp46**	67.5885479	0
**CD31**	60.6522302	0
**CD45**	46.7246424	0
**IgM**	45.2642992	0
**CD27**	44.2520023	0
**CD3**	40.4464091	0
**CD71**	36.7131408	0
**Ly6C**	32.3546739	0
**CD8a**	31.1428799	0
**CD11c**	26.8863317	0
**TCR**	18.3387956	0.00E+00
**CD11b**	15.014675	7.13E-243
**CD5**	15.0026102	1.24E-242
**Ly6G**	9.22206121	2.72E-130
